# Modification of carbonic anhydrase II with acetaldehyde, the first metabolite of ethanol, leads to decreased enzyme activity

**DOI:** 10.1186/1471-2091-9-32

**Published:** 2008-11-27

**Authors:** Fatemeh Bootorabi, Janne Jänis, Jarkko Valjakka, Sari Isoniemi, Pirjo Vainiotalo, Daniela Vullo, Claudiu T Supuran, Abdul Waheed, William S Sly, Onni Niemelä, Seppo Parkkila

**Affiliations:** 1 Institute of Medical Technology, Tampere University Hospital, 33520 Tampere, Finland; 2 School of Medicine, University of Tampere and Tampere University Hospital, 33520 Tampere, Finland; 3 Department of Chemistry, University of Joensuu, 80101 Joensuu, Finland; 4 Università degli studi di Firenze, Laboratorio di Chimica Bioinorganica, I-50019 Sesto Fiorentino (Firenze), Italy; 5 Edward A. Doisy Department of Biochemistry and Molecular Biology, Saint Louis University School of Medicine, St. Louis, 63104 Missouri, USA; 6 Department of Laboratory Medicine and Medical Research Unit, Seinäjoki Central Hospital and University of Tampere, 60220 Seinäjoki, Finland

## Abstract

**Background:**

Acetaldehyde, the first metabolite of ethanol, can generate covalent modifications of proteins and cellular constituents. However, functional consequences of such modification remain poorly defined. In the present study, we examined acetaldehyde reaction with human carbonic anhydrase (CA) isozyme II, which has several features that make it a suitable target protein: It is widely expressed, its enzymatic activity can be monitored, its structural and catalytic properties are known, and it contains 24 lysine residues, which are accessible sites for aldehyde reaction.

**Results:**

Acetaldehyde treatment in the absence and presence of a reducing agent (NaBH_3_(CN)) caused shifts in the pI values of CA II. SDS-PAGE indicated a shift toward a slightly higher molecular mass. High-resolution mass spectra of CA II, measured with and without NaBH_3_(CN), indicated the presence of an unmodified protein, as expected. Mass spectra of CA II treated with acetaldehyde revealed a modified protein form (+26 Da), consistent with a "Schiff base" formation between acetaldehyde and one of the primary NH_2 _groups (e.g., in lysine side chain) in the protein structure. This reaction was highly specific, given the relative abundance of over 90% of the modified protein. In reducing conditions, each CA II molecule had reacted with 9–19 (14 on average) acetaldehyde molecules (+28 Da), consistent with further reduction of the "Schiff bases" to substituted amines (N-ethyllysine residues). The acetaldehyde-modified protein showed decreased CA enzymatic activity.

**Conclusion:**

The acetaldehyde-derived modifications in CA II molecule may have physiological consequences in alcoholic patients.

## Background

Acetaldehyde, a product of ethanol metabolism, has been suggested to play a pivotal role in the toxicity of ethanol to human tissues [1-3]. It can form covalent, stable or unstable adducts with amino acids and nucleophilic biomolecules [4-10]. The adduct formation may result in changes in the physicochemical properties of proteins, nucleic acids, and lipids, disturb normal cellular functions, and create adverse immunological responses [8,11-15].

**Figure 1:**
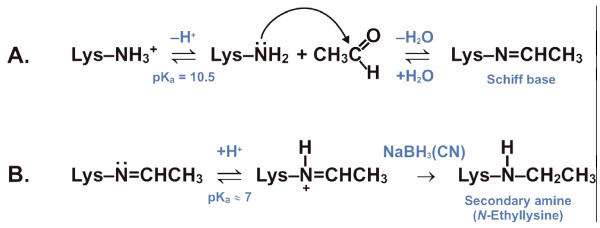
**The reaction mechanism of lysine residues with acetaldehyde, in the absence (A) and presence (B) of a reducing agent NaBH_3_(CN)**.

Aldehyde incorporation can lead to the formation of several different types of protein modifications, which have been previously identified in *in vitro *studies and in studies on alcohol abusers *in vivo *[6,8,14,16,17]. Acetaldehyde reacts primarily with reactive lysine residues of preferred target proteins [18-25]. It appears that under appropriate reducing conditions, proteins with abundant amounts of reactive lysine residues are modified at acetaldehyde concentrations that may be present in tissues after alcohol intake [8,25-28]. Figure 1 depicts the common reaction mechanism of the side chain amino groups (ε-NH_2_) of lysine residues with acetaldehyde, in the absence (A) and presence (B) of a reducing agent NaBH_3_(CN). In the former case, an unstable adduct (a "Schiff base") is formed, while in the latter case a "Schiff base" is further reduced to form a stable adduct (an N-ethyllysine residue). In the absence of reducing agents, stable cyclic imidazolidinone structures are also formed in a reaction between acetaldehyde and the free α-amino group of the aminoterminus of haemoglobin [16,20,29,30]. Previous studies have identified adducts with erythrocyte membrane proteins [31], haemoglobin [5,20,32-34], albumin, transferrin and lipoproteins [14,35,36], tubulin [21], ethanol-metabolizing cytochrome P450IIEI enzyme [37], collagens [38], and ketosteroid reductase [39].

In the present study, we used carbonic anhydrase (CA) isozyme II as a model to investigate the effects of acetaldehyde reaction with the protein. CA II is a well characterized enzyme expressed in several organs, including the brain, stomach, gut, kidney, and reproductive organs, and it is highly abundant in erythrocytes [40]. It is one of the most efficient enzymes known in the animal kingdom, catalyzing the reversible hydration of carbon dioxide at a rate of 1.4 × 10^6 ^molecules per second [41]. CA II was our choice of model protein, because its structural and catalytic properties are well known and it contains 24 lysine residues (out of 260 amino acids), each a potential site for acetaldehyde reaction. Currently, there are also valid methods to monitor CA II catalytic activity to assess the functional consequences of acetaldehyde reaction. Importantly, CA II catalytic activity is essential for several physiological processes such as gastric acid formation, alkalization of pancreatic juice and bile, renal proton secretion, bone resorption, and cerebrospinal fluid secretion [40]. Although proteomic *in vivo *evidence of this modification is still lacking, one would expect a wide variety of adverse effects in these physiological processes, if CA II activity was disturbed due to acetaldehyde reaction in alcoholic patients.

## Methods

### Production of recombinant human CA II

The recombinant human CA II enzyme was produced in *E. coli *[42] and purified to homogeneity using CA inhibitor affinity chromatography as described in [43].

### Labelling of CA II with acetaldehyde

Human blood samples and recombinant human CA II enzyme were treated with various concentrations of acetaldehyde either in the presence or absence of a reducing agent, NaBH_3_(CN). All reagents were maintained and pipeting was performed at +4°C to minimize acetaldehyde evaporation. The sample tubes containing 1/10 diluted (in H_2_O) blood or CA II enzyme with or without acetaldehyde in H_2_O were tightly sealed and incubated at 37°C for 2 hr. Then 10 mM NaBH_3_(CN) or equal volume of H_2_O was added to each sample tube, and the incubation at +37°C was continued for 22 hr. After the incubation the samples were quickly cooled down to +4°C.

### Isoelectric focusing and SDS-PAGE

IEF was carried out using Novex Pre-Cast vertical IEF gels (pH 3–10) (Invitrogen, Carlsbad, CA) containing 2% ampholytes. One μg samples of recombinant CA II protein untreated or treated with 10 mM NaBH_3_(CN) and various acetaldehyde concentrations were applied to each lane. The electrophoreses were performed in an Xcell SureLock™ Mini-Cell unit (Invitrogen) at a constant power of 2 W per gel for 2 hr with a voltage limit of 500 V. The polypeptides were visualized using Colloidal Blue staining kit (Invitrogen).

All the reagents for SDS-PAGE were from Invitrogen except for the protein markers that were obtained from Bio-Rad Laboratories (Richmond, CA). The electrophoreses were performed in an Xcell SureLock™ Mini-Cell unit (Invitrogen) under reducing conditions, using NuPAGE™ 10% Bis-Tris gels. The polypeptide bands were stained using Colloidal Blue staining kit (Invitrogen).

### CA activity measurements

CA catalytic activity was first determined using a slightly modified end-point titration method of Maren et al. [44,45]. Briefly the steps included: 150 ng of human CA II or 1 μl of 1/10 diluted blood sample was added to 500 μl of ice-cold assay buffer (20 mM imidazole, 5 mM Tris (BASE), 0,2 mM *p*-nitrophenol). The cuvette containing the sample and assay buffer was placed in a Lambda 35 UV/VIS (Perkin Elmer Instruments, Waltham, MA) spectrophotometer and 500 μl of ice-cold CO_2_-saturated H_2_O was added into the cuvette. The exact time for the yellow color disappearance was counted. In the control experiments without the CA II enzyme, the color disappeared in 50 sec. Statistical significance of the enzyme activity results was assessed using Student's t test and was denoted as p values.

The enzymatic activities of native recombinant human CA II and the enzyme treated with 100 mM acetaldehyde under reducing conditions were also assayed using an Applied Photophysics (Leatherhead, UK) stopped-flow instrument. Reaction was measured using 0.2 mM phenol red as an indicator, in 10 mM Hepes, 0.1 M Na_2_SO_4_, pH 7.5, for a period of 10–100 sec. To determine the kinetic parameters, CO_2 _concentration ranged from 1.7 to 17 mM. Kinetic parameters were obtained from Lineweaver-Burk plots, as reported earlier, and represent the mean from at least three different determinations.

### Sample preparation for mass spectrometry

Due to a low tolerance of ESI for high salt concentrations, protein samples were first desalted using a PD-10 column (Amersham-Biosciences, Billingham, UK) equilibrated in advance with 10 mM ammonium acetate (pH 6.8) buffer and ten 1-mL fractions were collected. Fractions 3–5 were combined and concentrated to ~500 μL using an Ultrafree-0.5 (10-kDa cut-off) centrifugal filter device (Millipore, Billerica, MA, USA). Protein concentrations were determined by absorbance at 280 nm using a calculated extinction coefficient of ε_280 _= 50070 M^-1 ^cm^-1^. For mass spectrometry, samples were further diluted with acetonitrile/water/acetic acid (49.5:49.5:1.0, v/v) solution to an approximate concentration of 5 μM.

### Mass spectrometry

All experiments were performed on a 4.7-T Bruker BioAPEX-Qe Fourier transform ion cyclotron resonance (FT-ICR) mass spectrometer (Bruker Daltonics, Billerica, MA, USA), interfaced to an external Apollo-II™ electrospray ionization (ESI) source. Protein samples were directly infused at a flow rate of 1.5 μL min^-1^. ESI-generated ions were externally accumulated in an RF-hexapole ion trap for 400 ms and transmitted through a high-voltage optics region to an Infinity ICR cell for "sidekick" trapping, conventional "RF-chirp" excitation and broadband detection. For each spectrum, a total of 256 co-added (512-kWord) time-domain transients were zero-filled once prior to fast Fourier transform and magnitude calculation. Frequency-to-*m*/*z *calibration was performed externally with respect to the ions of an ES Tuning Mix (Agilent Technologies, Santa Clara, CA, USA) calibration mixture. All data were processed using Bruker XMASS 7.0.8 software.

## Results

### Isoelectric focusing and SDS PAGE

**Figure 2:**
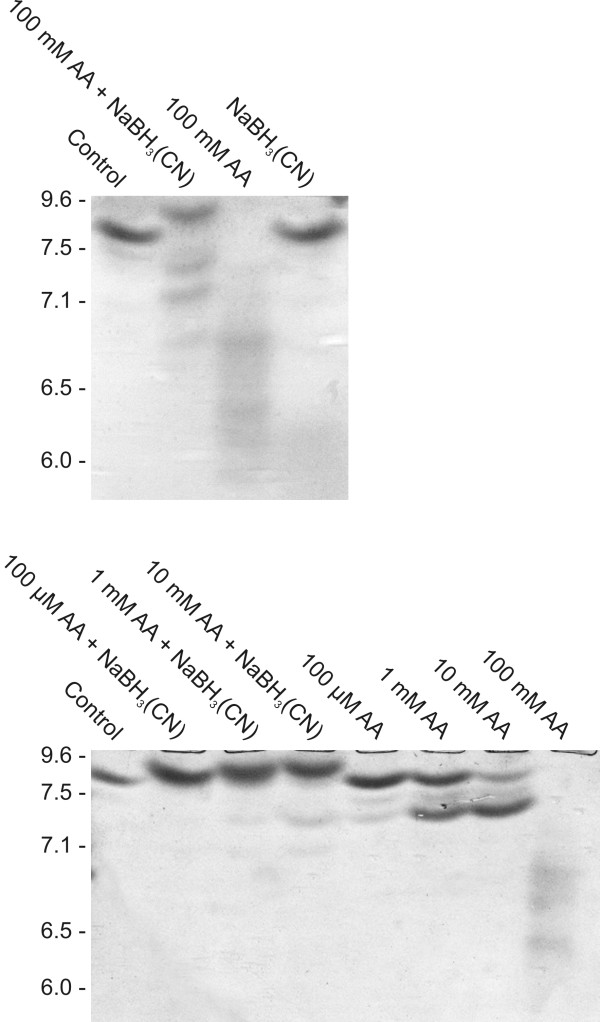
**Isoelectric focusing of human recombinant CA II treated with various concentrations of acetaldehyde (AA) in the presence or absence of a reducing agent, 10 mM NaBH_3_(CN)**.

Recombinant human CA II treated with various concentrations of acetaldehyde was subjected to isoelectric focusing (Fig. 2, upper panel). The pI value of untreated enzyme was about 7.9. Acetaldehyde treatment in the presence or absence of a reducing agent NaBH_3_(CN) (sodium cyanoborohydride) caused shifts in the pI values. In the presence of the reducing agent, the isoelectric point became slightly more basic after acetaldehyde treatment. The results shown in Fig. 2 (lower panel) showed a concentration dependent shift to lower pI with increased aldehyde concentrations. Even 100 μM acetaldehyde caused a visible change in the charge of the CA II molecule. Bands with pI values of 6.8, 7.1 and 7.3 were identified with increasing acetaldehyde concentrations. Without NaBH_3_(CN), all changes in isoelectric points were toward more acidic values. Acetaldehyde concentrations of 100 μM-10 mM produced two extra bands of pI values at 7.3 and 7.4. At 10 mM concentration, the 7.3 pI value became most prominent. Surprisingly, the pattern was completely different when acetaldehyde reached 100 mM concentration. The protein appeared as a smear with three faint bands of 6.4, 6.7 and 6.9 pI values. It is notable that NaBH_3_(CN) alone had no effect on the isoelectric point of CA II (Fig. 2, upper panel).

**Figure 3:**
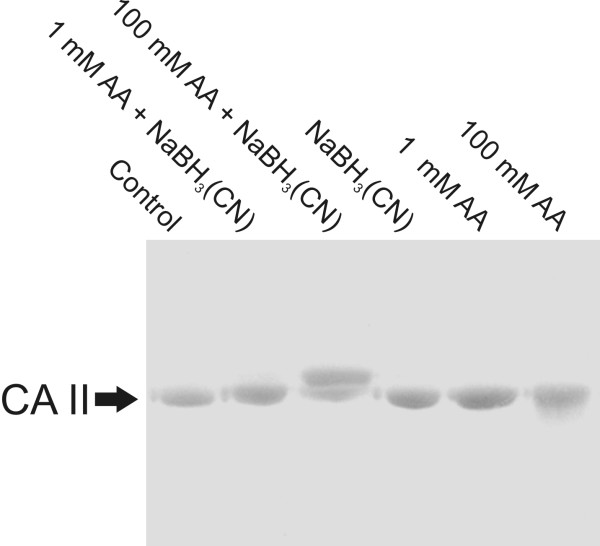
**SDS-PAGE of human recombinant CA II treated with acetaldehyde (AA)**. The acetaldehyde concentrations are shown in the figure. Some of the samples were subjected to reducing conditions using NaBH_3_(CN).

SDS PAGE was performed on acetaldehyde-treated CA II samples. The results in Figure 3 demonstrate that 100 mM acetaldehyde in the presence of NaBH_3_(CN) produced a shift toward a slightly higher molecular mass. In contrast, the same acetaldehyde concentration without the reducing agent produced a faint smear, which could indicate a partial degradation of the protein or it may be a consequence of unwanted interactions between the ampholytes and acetaldehyde in a medium.

### Kinetic analyses

**Figure 4:**
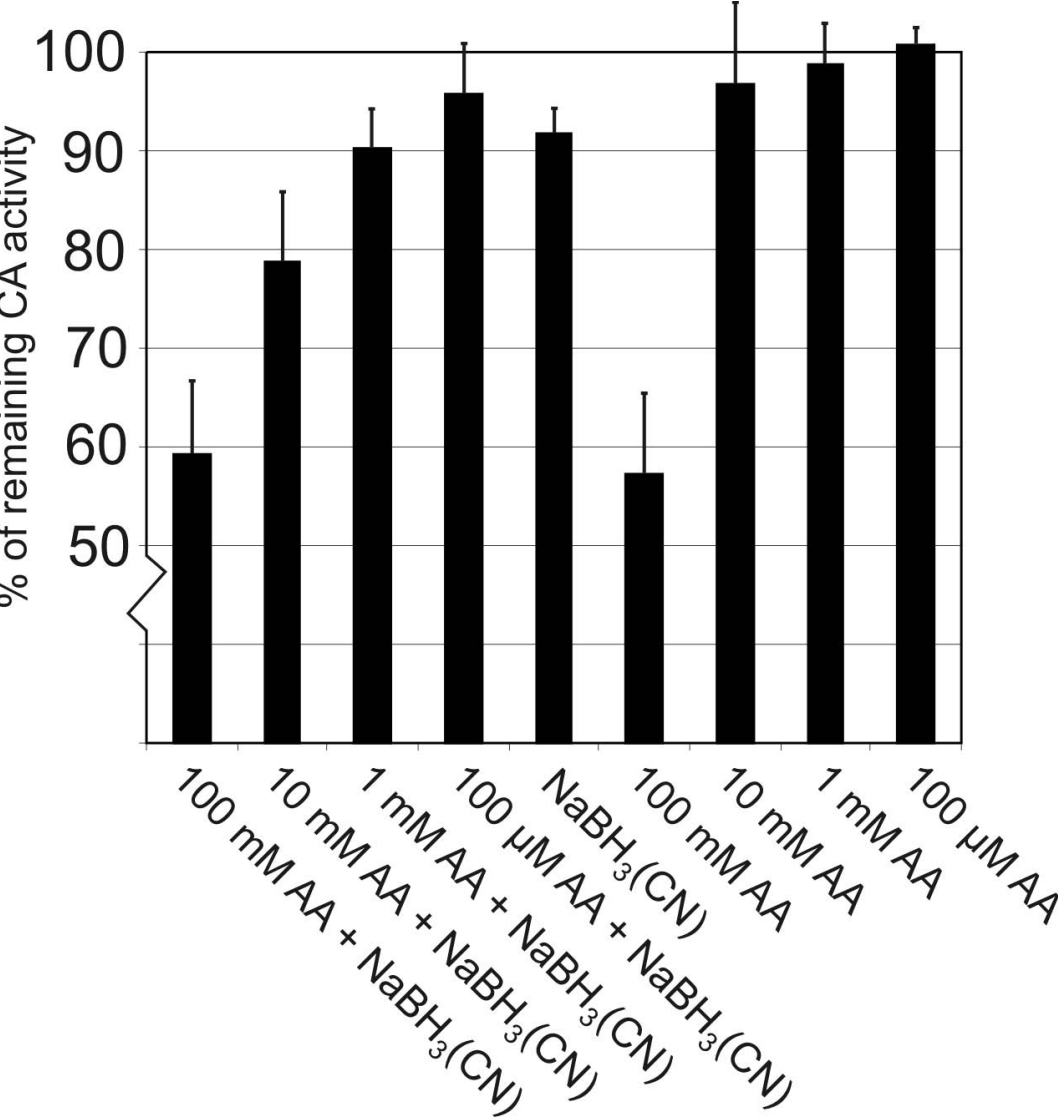
**Carbonic anhydrase activity of human blood samples treated with various concentrations of acetaldehyde (AA) in the presence or absence of a reducing agent, 10 mM NaBH_3_(CN)**. The activity assay was performed using a previously described assay method of Maren [44,45]. The obtained values from three assays are indicated as mean +/- standard deviation.

**Figure 5:**
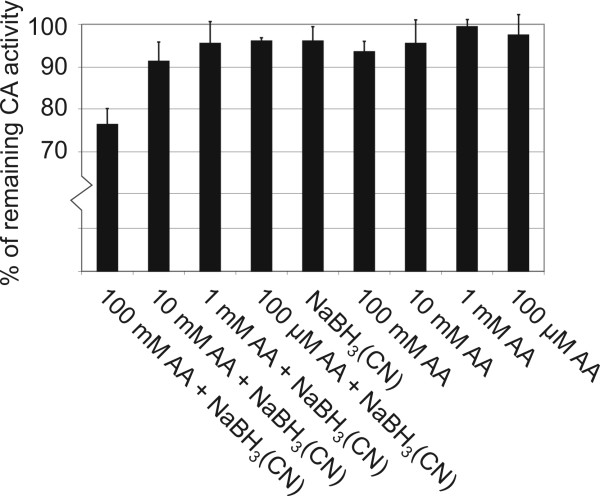
**Carbonic anhydrase activity of human recombinant CA II treated with various concentrations of acetaldehyde (AA) in the presence or absence of a reducing agent, 10 mM NaBH_3_(CN)**. The activity assay was performed using a previously described assay method of Maren [44,45]. The obtained values from three assays are indicated as mean +/- standard deviation.

CA activity measurements were performed using a previously described assay method of Maren [44,45] combined to a spectrophotometric color determination. First, we determined the CA activities of blood samples treated with acetaldehyde. Figure 4 demonstrates that 100 mM acetaldehyde produced over 40% reduction in CA activity both in the presence (p = 0.016) and absence (p = 0.012) of NaBH_3_(CN). A 10 mM concentration of acetaldehyde also reduced the CA activity in the presence of NaBH_3_(CN), but this change did not reach statistical significance (p = 0.052). In the second set of experiments, we measured enzymatic activities of CA II samples in analogous conditions as described above. Acetaldehyde (100 mM) in the presence of NaBH_3_(CN) caused 23.7% reduction in CA II activity (p = 0.001)(Fig. 5). In the absence of NaBH_3_(CN), the activity was only slightly reduced (p = 0.045). All other changes observed were non-significant (p > 0.05).

The enzyme activity data obtained from the stopped-flow analysis are shown in Table 1. This method indicated that the acetaldehyde-modified enzyme is about one-third as active as the native CA II (considering the specificity constant k_cat_/K_m_). On the other hand, the inhibition constant of acetazolamide is about three times higher for the modified protein compared to the native one.

**Table 1:** Kinetic and inhibitory properties of CA II measured by the stopped-flow method

**Enzyme**	**k_cat _(s^-1^)**	**K_M _(mM)**	**k_cat_/K_M _(M^-1^s^-1^)**	**K_I _(acetazolamide) (nM)**
**Native CA II**	1.40·10^6^	9.3	1.5·10^8^	12
**CA II + AA + NaBH_3_(CN)**	0.77·10^6^	9.3	0.5·10^8^	35

### Mass spectrometry

**Figure 6:**
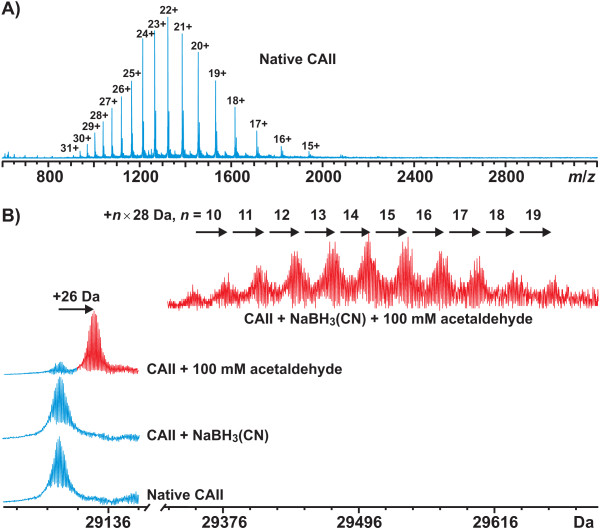
**ESI FT-ICR mass spectra of human recombinant CA II**. (A) A broadband mass spectrum of native CA II (different protein ion charge states have been assigned as *z *+ = [CA II + *z*H]^*z*+^) and (B) an expanded view on the charge state 24+ for native CA II, and CA II samples treated with 100 mM acetaldehyde in the absence and presence of NaBH_3_(CN). The determined masses have been indicated. In panel B, the mass scale in Da is set for the charge state 24+.

Figure 6A presents an ESI FT-ICR mass spectrum measured for native CA II. A protein ion charge state distribution from 13+ to 32+ representing apo-protein (i.e., protein without a bound Zn^2+ ^cation) was observed. The most abundant isotopic mass (*m*_m.a._) of the protein was determined to be 29098.07 ± 0.07 Da which agrees well with the calculated value (*m*_m.a. _= 29097.93 Da for CAH2_HUMAN (Swiss-Prot entry P00918), with the initial methionine removed and the second residue changed from serine to alanine due to the used cloning strategy).

Figure 6B presents an expanded view on the charge state 24+ of the mass spectra of native CA II (Figure 6A) as well as CA II incubated in the presence of NaBH_3_(CN), acetaldehyde (AA), and both. In the presence of NaBH_3_(CN), the mass spectrum was identical to that observed for native CA II. In contrast, when reacted with 100 mM acetaldehyde in the absence of NaBH_3_(CN), the mass of CA II increased by ~26 Da (*m*_m.a. _= 29124.09 ± 0.09 Da), consistent with a "Schiff base" formation between acetaldehyde and a primary amino group, e.g., ε-NH_2 _of lysine (Δ*m*_theor _= +26.02 Da). This reaction was highly specific given that the modified protein form was present for as much as 90% as compared to the unmodified form. In the presence of NaBH_3_(CN), a more drastic change was observed. Evenly distributed new signals appeared in the mass spectrum, with a mass increase of ~*n *× 28 Da, *n *ranging from 9 to 19 (for the three most abundant forms, *m*_m.a. _= 29462.51 ± 0.06 Da (*n *= 13), 29490.54 ± 0.05 (*n *= 14), and 29518.59 ± 0.05 Da (*n *= 15)). This is consistent with the formation of multiple stable covalent acetaldehyde adducts with CA II, most probably *N*-ethyllysine residues (Δ*m*_theor _= +28.03 Da).

## Discussion

Acetaldehyde has been demonstrated to be able to bind to several different proteins, both *in vitro *and *in vivo*. The structural modifications due to acetaldehyde binding may affect the conformation, acid-base properties, and/or hydrogen-bonding patterns of amino acids on the surface or within the active site of an enzyme, thereby disrupting the normal protein function. Although several lines of previous investigations during the last two decades have focused on the generation of acetaldehyde adducts, it has turned out to be a great challenge to explore the functional consequences of these acetaldehyde-induced modifications without proper functional assays. Acetaldehyde is a very reactive compound, and thus, it has been particularly difficult to determine the presence of unstable adducts formed under non-reductive conditions.

The present results showed that human CA II is a good target protein for acetaldehyde modification. It has a high number of lysine residues (24 in total) in its primary sequence. The highly sensitive mass spectrometric method indicated that, under reducing conditions, each CA II enzyme molecule had reacted with up to 19 (14 on average) acetaldehyde molecules to form stable covalent adducts (N-ethyllysine residues). Although such a high number of acetaldehyde was bound to the enzyme, the modified protein still retained much of the enzymatic activity. Using the modified Maren's method combined with a spectrophotometric analysis, we found that the enzyme activity decreased 23.7% under supraphysiological (100 mM) acetaldehyde concentrations in reducing conditions. The stopped-flow CA activity assay showed a significantly greater decrease in the enzyme activity when compared to the spectrophotometric assay. Even though these assay methods produced different rates for the acetaldehyde-induced inhibition, both techniques clearly indicated that acetaldehyde binding reduces the catalytic activity.

Interestingly, only a single acetaldehyde molecule had reacted with CA II under non-reductive conditions, forming the unstable covalent adduct (''Schiff base''). Due to high mass resolution inherent for the FT-ICR technique, unequivocal differentiation between the ''Schiff base'' (+26.02 Da) and the substituted amine (+28.03 Da), i.e. ~2-Da mass difference at 29 kDa, was possible (Figure 1). According to the mass spectrometry, over 90% of the enzyme molecules were modified with acetaldehyde. However, repeated measurements with the acetaldehyde-treated sample, after being stored two weeks at 4°C, indicated the presence of an unmodified CA II only, consistent with unstable character of the formed ''Schiff base''. To further localize the modification site, both native and acetaldehyde-treated CA II samples were subjected to on-line pepsin digestion prior to mass spectrometry. Although the resulting peptide maps had 100% sequence coverage, the observed peptides were markedly larger (up to 10 kDa) in the presence of acetaldehyde, possibly due to decreased protease activity, making spectral assignments occasionally ambiguous. However, two of the observed 6.3-kDa peptic peptides had a mass difference of 26.01 Da, suggesting the modification site within the first 56 residues. Owing to the experimental difficulties in obtaining good tandem mass spectra for these large peptic peptides, more experiments with different proteases are warranted for the further identification of the modification site in CA II.

Both SDS-PAGE and isoelectric focusing suggested that 100 mM acetaldehyde might induce degradation of CA II protein under non-reductive conditions. However, mass spectrometry did not show any evidence for the accelerated degradation of CA II (i.e., no change in the absolute intensity of the intact protein or evidence of the peptide signals), suggesting that the smear observed in isoelectric focusing may have resulted from chemical interactions between ampholytes and acetaldehyde under non-reductive conditions.

More detailed structural studies are needed to characterize the acetaldehyde-induced modifications of CA II. This enzyme, although only weakly expressed in liver [46,47], represents an excellent model for both structural and functional studies of acetaldehyde modification. Its crystal structure and kinetic properties have been reported by several groups [48-53]. These studies can be expanded to other CA isozymes that are more highly expressed in the liver, including CA III, VA, and CA XIV [54-56]. These enzymes may also be susceptible to modification because acetaldehyde can reach over 100 μM concentration in the liver of alcoholic patients [57], even though concentrations are lower, though still in a micromolar range, in the blood [58,59].

Accurate acetaldehyde determination from biological samples has been extremely challenging. It is widely accepted that single doses of ethanol do not significantly increase blood free acetaldehyde concentrations [60,61]. Nonetheless, such doses may elevate acetaldehyde levels within intracellular compartments or cell membranes. Baraona et al. [62] tested the blood of 5 healthy individuals and 6 alcoholic patients and showed that most of the blood acetaldehyde was present in the erythrocytes after alcohol consumption. *In vivo*, acetaldehyde concentration within the erythrocytes is about 3–10 times higher than in the plasma [62,63]. Although acetaldehyde concentrations are probably highest in the liver, the site of ethanol metabolism, acetaldehyde modified protein epitopes have been also located to other organs and cell types. Positive immunohistochemical staining for acetaldehyde adducts has been demonstrated in the brain, heart, skeletal muscle, and erythrocytes [14,64-66]. All of these tissues and cells contain several CA isozymes that may become functionally impaired because of adduct formation. Therefore, these modifications deserve further study to determine the exact submolecular defects caused by acetaldehyde in each isozyme.

## Conclusion

In the present study, we showed that acetaldehyde, the first metabolite of ethanol, can modify the ubiquitous enzyme, carbonic anhydrase. Mass spectrometric analysis indicated that one of the primary NH_2 _groups (e.g., in lysine side chain) in the CA isozyme II had reacted with acetaldehyde under non-reducing condition, consistent with a "Schiff base" formation. In reducing conditions, each CA II molecule had reacted with 9–19 (14 on average) acetaldehyde molecules, consistent with further reduction of the "Schiff bases" to substituted amines (N-ethyllysine residues). The latter structural change led to decreased enzyme activity, which may have important physiological consequences in alcohol abusers.

## Authors' contributions

ON, JV, JJ and SP designed the study. AW and WSS provided the recombinant E. coli bacteria for CA II production. FA purified the recombinant CA II protein. FA, DV, CTS, and SP carried out the enzyme activity assays. JJ, SI, JV and PV performed the mass spectrometry. FA, ON, JV, JJ, CTS and SP drafted the manuscript. All authors read and approved the final manuscript.
